# Aberrant Expression Levels of Androgen Receptor and SRD5A2 in Epididymal Epithelial Cells of Crossbred Infertile Cattle–Yak

**DOI:** 10.3390/ani15050660

**Published:** 2025-02-24

**Authors:** Manita Wittayarat, Kimika Kawanishi, Haruka Ohata, Megumi Nagahara, Rentsenkhand Sambuu, Otgonjargal Sambuu, Maki Hirata, Fuminori Tanihara, Masayasu Taniguchi, Takeshige Otoi, Yoko Sato

**Affiliations:** 1 Faculty of Veterinary Science, Prince of Songkla University, Songkhla 90110, Thailand; mwittayarat@gmail.com; 2 Department of Medical Engineering, Faculty of Allied Sciences, University of East Asia, Yamaguchi 751-8503, Japan; 3 Bio-Innovation Research Center, Tokushima University, Tokushima 779-3233, Japan; nagahara@tokushima-u.ac.jp (M.N.); mhirata@tokushima-u.ac.jp (M.H.); tanihara@tokushima-u.ac.jp (F.T.); otoi@tokushima-u.ac.jp (T.O.); 4 Institute for Extension of Agricultural Advanced Technology, Ulaanbaatar 17024, Mongolia; 5 School of Veterinary Medicine, Mongolian University of Life Sciences, Ulaanbaatar 17024, Mongolia; otgonjargal.s@muls.edu.mn; 6 Department of Animal Reproduction, Joint Faculty of Veterinary Medicine, Yamaguchi University, Yamaguchi 753-0841, Japan; masa0810@yamaguchi-u.ac.jp

**Keywords:** androgen receptor, SRD5A, cattle–yak, hybrid, epididymis, yak

## Abstract

Although yaks and cattle belong to the same *Bovinae* subfamily and have the same number of chromosomes, hybrid males are sterile because of the inactivation or abnormality of gene expression related to the production of healthy normal sperm. Recently, the analysis of gene expression not only in the testis but also in the epididymis has offered hints about the mechanism of infertility, because the epididymis supports the maturation of sperm in acquiring the capacity of fertilisation. Sperm maturation processes have been thought to be androgen-dependent, and the androgen receptor (AR) can be activated by dihydrotestosterone converted from plasma testosterone by the 5α-reductase isoform 2 (SRD5A2) in epididymal cells. In the present study, we investigated the immuno-expression levels of the AR and SRD5A2 in the epithelial cells of the hybrid cattle–yak epididymal caput in comparison with yak samples using image analysis. Epididymal tissues from yaks (1–3 years of age) and hybrid cattle–yaks (2 years of age) were used in this study. In yaks, AR signal intensity did not show any changes in epididymal epithelial cells during maturation. However, in 2-year-old hybrid cattle–yaks, AR signal intensity was significantly higher in the principal cells of the epididymis compared to that of yaks of the same age, indicating that hybrid sterility is not likely related to AR deficiency in the epididymal epithelium. On the other hand, SRD5A2 signal intensity was stable during maturation in the epithelial cells of the yak epididymis. However, the epididymal SRD5A2 signal intensity in the epithelial cells of the hybrid cattle–yak was lower than that of the yak. This suggests that a deficiency in SRD5A2 production in the epididymis may result in hybrid infertility, as it can subsequently cause incomplete AR signal transduction and altered spermatozoa physiology.

## Simple Summary

This study explores the infertility observed in hybrid male cattle–yaks, focusing on the roles of the androgen receptor (AR) and 5α-reductase isoform 2 (SRD5A2) in the epididymis. Although both species belong to the same subfamily and share chromosome numbers, hybrid males are sterile due to abnormalities in sperm production. Recent analyses of gene expression in both the testis and epididymis have provided insights into the mechanisms underlying this infertility. The maturation of sperm, which is crucial for fertilisation, is believed to be dependent on androgens, with the AR being activated by dihydrotestosterone, a derivative of testosterone processed by SRD5A2 in epididymal cells. This research involved comparing the immuno-expression levels of the AR and SRD5A2 in the epididymal caput of yaks and hybrid cattle–yaks. The findings revealed that in yaks, AR signal intensity remained stable during maturation, while in hybrid cattle–yaks, AR signal intensity was significantly higher in principal cells compared to yaks of the same age. This indicates that AR deficiency is not the cause of hybrid sterility. Conversely, this study found that SRD5A2 signal intensity was stable during maturation in yak epididymal epithelial cells, whereas hybrid cattle–yaks exhibited lower SRD5A2 levels in cells compared to yaks of the same age. This deficiency in SRD5A2 production is suggested to contribute to hybrid infertility by impairing AR signal transduction and disrupting sperm physiology.

## 1. Introduction

Cattle–yak, a crossbreed between yak *(Bos grunniens)* and cattle *(Bos taurus)*, is recognised as a valuable animal species because it exhibits positive adaptability to extremely harsh environments, such as cold and anoxic conditions, in the high-altitude mountainous areas of the Qinghai–Tibetan Plateau, and shows greater meat and milk production compared to yak [1,2]. Several studies have described that the major limitation on expanding cattle–yak populations is generally associated with male infertility in the first filial generation (F1); however, only a few studies have accounted for the molecular and cellular physiology of the testes and epididymis [1,3]. We previously reported the features of epigenetic changes and the balance between cell proliferation and apoptosis in hybrid cattle–yak testes, which might be major factors in the disruption of normal germ cell development [2,4]. However, we believe that more studies are required to better explore the mechanisms of infertility in cattle–yak males, as infertility is a result not only of testicular defects, but also of epididymal defects.

The fertility of backcrossed hybrid offspring gradually recovers with each generation, but F2 male hybrids only occasionally produce a few dead sperm, though some sperm produced by F3 male hybrids show not only abnormality but also some normality [5]. There may be defects in the sperm maturation process in the epididymis in addition to the spermatogenesis process in the testis in hybrid cattle–yak. The epididymis is a duct-like organ in which spermatozoa undergo maturation, acquire motility, and gain the ability to fertilise the egg [6]. During development, the epididymis undergoes significant structural changes, including epithelial cell differentiation into principal, narrow, basal, and halo cells [7] as well as functional changes related to region-specific gene expression [8]. The epididymis has three portions (caput, corpus and cauda) with different functions, and the caput is thought to be important for the sperm maturation process [9]. Recent studies on the physiological roles of microRNAs (miRNAs) in the epididymis have shown that putative miRNAs are involved in male cattle–yak infertility via alterations in gene expression [1,3,10]. This suggests that infertility in hybrid cattle–yaks might be due to multiple genetic defects, some of which have not yet been elucidated.

Changes in the androgen receptor (AR) and its expressed proteins are among the causes of impaired spermatogenesis and idiopathic male infertility, as the AR plays a key role in androgen action [11,12]. Androgens such as testosterone or its metabolite 5α-dihydroxytestosterone (DHT) can act differently depending on the level of AR expression in various tissues or cells, and ARs are predominantly detected in the nuclei of testicular cells and in the epithelial and stromal cells of the epididymis [13]. The testosterone metabolite DHT, the active androgen catalysed by 5α-reductase isoforms 1 (SRD5A1) and 2 (SRD5A2), can bind to the AR and change AR conformation in the same manner as testosterone in order to either promote or suppress the expression of specific target genes [14,15]. SRD5A2 expression is predominant in the reproductive tissues, genital skin, and epididymis [16]. Although our previous work showed that the irregular expression of the AR and SRD5A2 in testicular Leydig cells may be associated with cattle–yak infertility [14], we performed this study to evaluate the expression and significance of the AR and 3β-HSD in the epithelial cells of the hybrid cattle–yak epididymis and to determine their correlation with infertility in comparison with yak samples, because the sperm maturation processes in the epididymis are highly related to androgen responses.

## 2. Materials and Methods

### 2.1. Animal and Sample Collection

All animal and sample collection procedures were reviewed and approved by the Animal Ethics Committee/Institutional Review Board of the University of East Asia (#AEC-TOUA-H30-4). The experimental yaks and cattle were obtained from yak farms in the Khövsgöl Province and Ikhtamir District of Arkhangai Province, Mongolia, during the breeding season in July 2012. Backcrossing between the sire yak and the first generation (F1) of female cattle yaks was used to generate a second generation (F2) of hybrid cattle–yaks. Testicular and caput epididymal samples from yaks of different ages (1-year-old, 2-year-old and 3-year-old) and 2-year-old F2 hybrid cattle–yaks (*n* = 3 in each group) were obtained by castration using an emasculator, knife, or slaughter. Three samples from each group were immediately fixed in 10% formalin in the field for 24 h and then transported to the laboratory for further processing.

### 2.2. Tissue Processing

The fixed tissues were rinsed with phosphate-buffered saline (PBS) three times for 1 h, dehydrated with serial alcohol concentrations (70–100%) for 1 h each, and then replaced with xylene three times for 1 h. Subsequently, the tissue samples were moved into xylene–paraffin (1:1) for 1 hr, and then they were impregnated in paraffin wax three times for 1 h before routine embedding. Serial sections (4 μm) were cut on a rotary microtome and mounted on slides. The developmental stages of the epididymis in each group were confirmed using haematoxylin and eosin staining.

### 2.3. Immunohistochemistry

All the procedures were performed at 25 °C. The sections were de-paraffinised with xylene, rehydrated using a descending graded ethanol solution, washed with PBS, and treated with 10 mM citric acid buffer (pH 6.0) for antigen retrieval in a microwave for 30 min. To prevent endogenous peroxidase activity, the sections were blocked with 0.3% hydrogen peroxide (H_2_O_2_) solution in methanol for 1 h. After the preincubation of the sections with normal goat immunoglobulin G (IgG, 500 μg/mL; Dako, Tokyo, Japan) dissolved in 1% bovine serum albumin (BSA; Sigma-Aldrich, Tokyo, Japan) in PBS for 1 h to block non-specific bindings, primary rabbit polyclonal antibody against the androgen receptor (AR) (N-20, 1:200; Santa Cruz Biotech, Santa Cruz, CA, USA) or 5α-reductase 2 (SRD5A2) (H-100, 1:100; Santa Cruz Biotech, CA, USA) diluted with 1% BSA in PBS was applied to the sections and incubated overnight. The negative control was a matched IgG isotype control for rabbits at the working concentration of each primary antibody. The sections were then incubated with goat anti-rabbit IgG (Fab’) and labelled with an amino acid polymer–peroxidase complex (Histofine Simple Stain MAX PO (R), Nichirei Co., Tokyo, Japan) for 30 min. Sections were visualised using 3-amino-9-ethylcarbazol (AEC) (Histofine Simple Stain AEC solution, Nichirei Co., Tokyo, Japan) for 15 min. The nuclei of the sections were counterstained with haematoxylin solution and mounted using an aqueous mounting medium (Nichirei Co., Tokyo, Japan) with a glass coverslip.

### 2.4. Semi-Quantification of Immunolabelling in Epididymis Epithelial Cells

All images were acquired using a microscope (Nikon Eclipse NiU-TRFLM; Nikon, Tokyo, Japan) equipped with a Nikon DS-Fi2-U3 digital camera (Nikon, Tokyo, Japan) attached to a computer. Examined fields were randomly selected by examiners who were blinded to the tissue groups. The semi-quantification of immunostained epididymis epithelial cells was performed as previously described by Sato et al. [14] with minor modifications. Briefly, the binarised images were subjected to optical density measurement using an automatic image analyser (NIS-Element D 4.3 I, Nikon, Tokyo, Japan) at the surface areas occupied by immunostained cells. The optical density data were then reversed to adjust for luminance intensity, and the signal intensity was measured and expressed in arbitrary units (U). Background intensities of the samples were quantified using the same method. The different samples were stained at the same time, and the same conditions were used for immunostaining including coloration time, with the images also being taken under the same light conditions. The measurement was repeated three times, and the average value for each sample was used for the comparison of groups.

AR immunostaining in the nucleus was measured by NIS-Element for optical density after the conversion of binarised images. The average optical density of the haematoxylin stain of the negative control was subtracted from the AR optical density. And then, those cells with a mean value of optical density above a defined threshold were counted as AR- immunopositive cells. The AR-immunopositive cell staining of principal and basal cells was determined from an average of ten fields per sample, which were visually counted. To assess the mean percentage of immunopositive cells, the number of immunopositive cells was divided by the total number of cells of the same cell type in the epididymal area.

For the semi-quantification of SRD5A2-immunostained epididymal epithelial cells, we enclosed 5 areas per field containing several epididymal cells, covering the area from the luminal side to the basal side, and subtracted their respective nuclear area from them to define the cytoplasmic area; then, we measured the optical density. The relative intensity of SRD5A2 in the epididymal epithelial cells was calculated using the following formula:

Relative intensity in epididymal epithelial cells = (mean intensity of SRD5A2 in each type of epididymal epithelial cell) − mean intensity of the background.

We set the mean value of 1-year-old yak expression as a reference to provide semi-quantitative data. The average arbitrary units (U) of cells labelled with each reagent per experiment were determined from the average of five fields per sample.

### 2.5. Statistical Analysis

All values were expressed as means ± SEMs. Student’s *t*-test was used to assess the differences between the data of 2-year-old yak and 2-year-old cattle–yaks using Microsoft Excel ver.16.88 (Microsoft Corp., Redmond, WA, USA). Analysis of variance (ANOVA) and Tukey’s multiple comparison tests were performed to investigate the differences between 1-, 2- and 3-year-old yaks using GraphPad Prism7 software ver.7.0e or JMP ver. 14.2 (SAS Institute Inc., Tokyo, Japan). Differences with probability values *p* ≤ 0.05 were considered statistically significant.

## 3. Results

### 3.1. AR Expression in Epididymal Epithelial Cells During Yak Development

**Figure 1:**
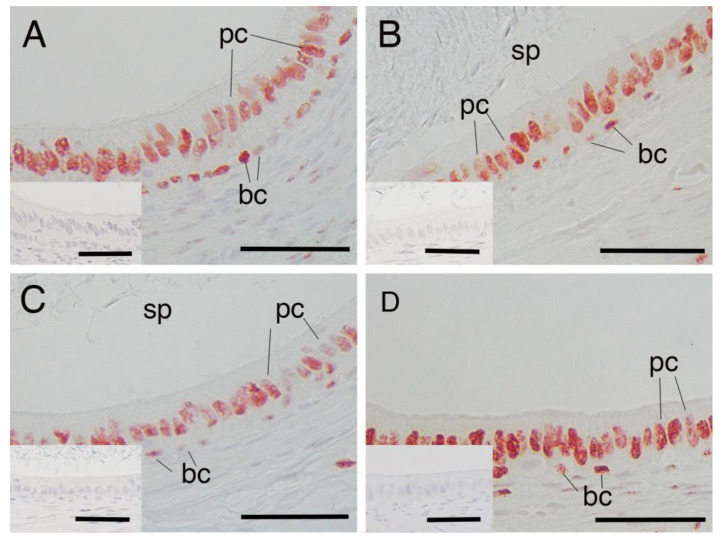
Expression of androgen receptor in epididymal cells of yak and cattle–yak. (**A**) immature yak (1 year old), (**B**) mature yak (2 years old), (**C**) mature yak (3 years old) and (**D**) crossbred cattle–yak (F2) (2 years old). pc—principal cell; bc—basal cell; sp—sperm. Insets—negative control. Scale bars—50 μm.

**Figure 2:**
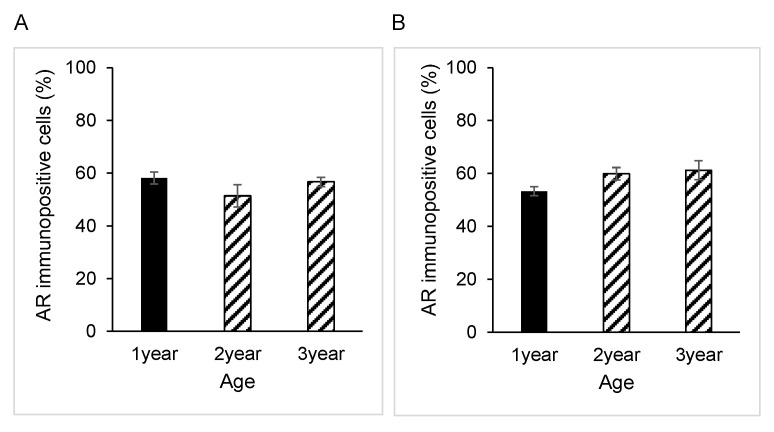
Developmental changes in androgen receptor expression in yaks of different ages. Percentage of androgen receptor-immunopositive epididymal cells: (**A**) principal cells and (**B**) basal cells. Bars and error bars represent means ± SEs (1 year, *n* = 3; 2 years, *n* = 3; 3 years, *n* = 3).

Immunostaining for AR was present in the nucleus of both principal and basal cells of the epididymis in all samples obtained from yaks of different ages (Figure 1A–C). However, there were no significant differences between the percentages of AR-immunopositive cells in either principal or basal cells among the age groups of 1, 2 and 3 years (Figure 2A,B). The negative control did not show any staining (Figure 1A–C insets).

### 3.2. AR Expression in Epididymal Epithelial Cells of Mature Yak and Cattle–Yak

**Figure 3:**
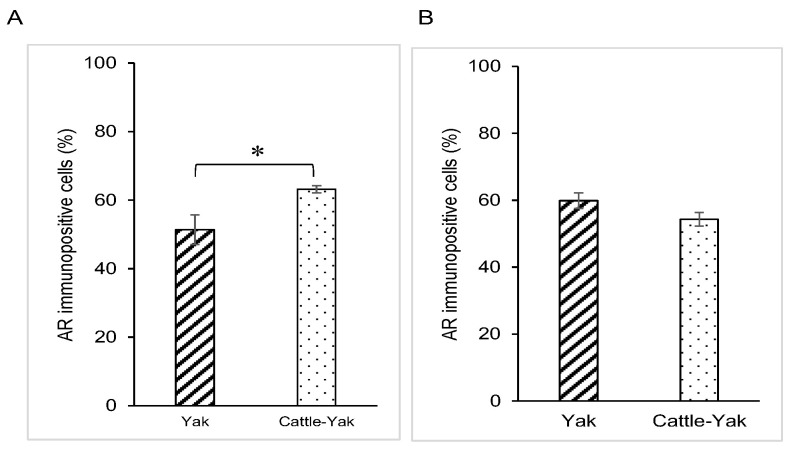
Comparison of androgen receptor expression changes in 2-year-old yaks and 2-year-old crossbred cattle–yaks (F2). Percentage of androgen receptor-immunopositive epididymal cells: (**A**) principal cells and (**B**) basal cells. Bars and error bars represent means ± SEs (yak, *n* = 3; F2, *n* = 3). Asterisk represents significant differences (*p* < 0.05).

AR expression was also detected in the nucleus of the epithelium in both principal cells and basal cells of the epididymis of 2-year-old cattle–yaks (Figure 1D). The percentage of AR-immunopositive principal cells in the 2-year-old cattle–yak epididymis was significantly higher than that in 2-year-old yaks (*p* < 0.05) (Figure 3A). However, no significant differences in AR expression levels in basal cells of the epididymis were detected between 2-year-old yaks and cattle–yaks (Figure 3B). The negative control did not show any staining (Figure 1A,D insets).

### 3.3. SRD5A2 Expression in Epididymal Epithelial Cells During Yak Development

**Figure 4:**
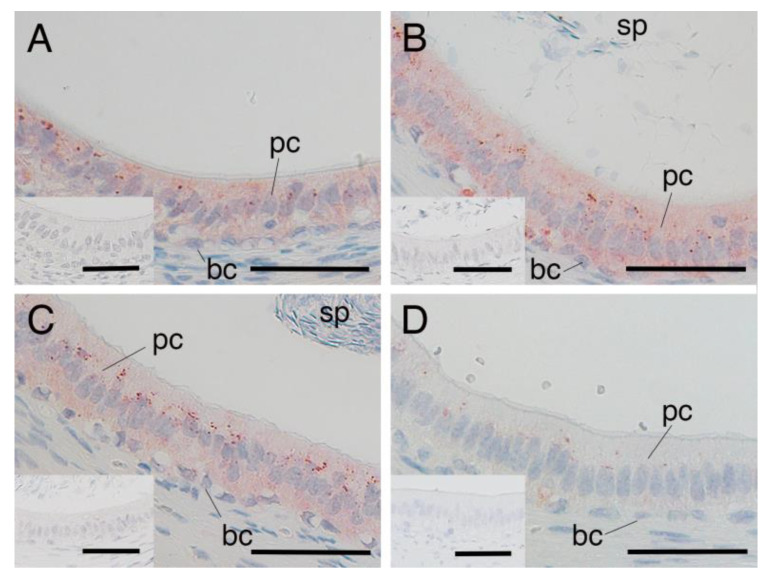
Expression of 5α-reductase2 in epididymal cells of yaks and cattle–yaks: (**A**) immature yak (1 year old), (**B**) mature yak (2 years old), (**C**) mature yak (3 years old) and (**D**) crossbred cattle–yak (F2) (2 years old). pc—principal cell; bc—basal cell; sp—sperm. Insets—negative control. Scale bar—50 μm.

**Figure 5:**
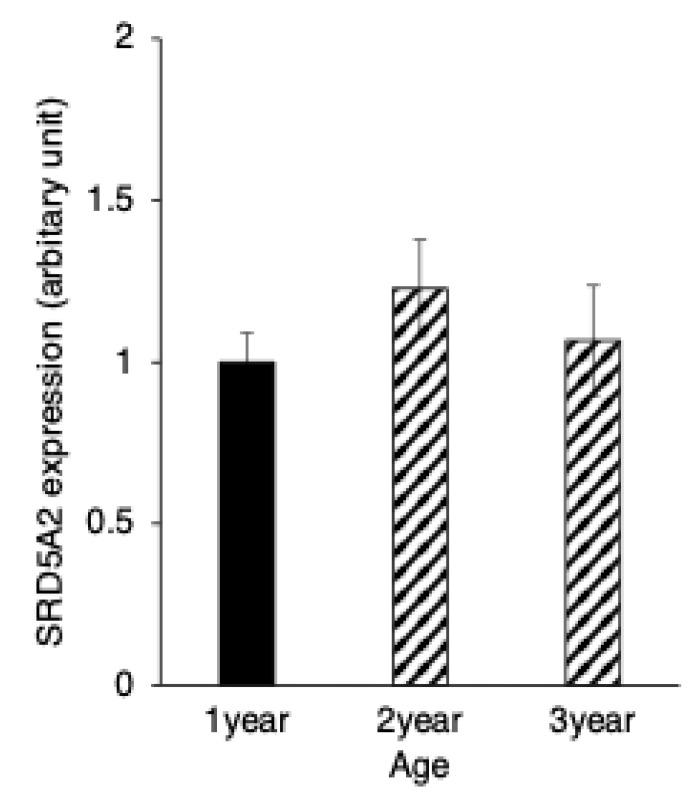
Developmental changes in 5α-reductase2 expression in yaks of different ages. 5α-reductase2 expression levels in immunopositive epididymal cells. Bars and error bars represent means ± SEs (1 year, *n* = 3; 2 years, *n* = 3; 3 years, *n* = 3).

SRD5A2 immunostaining was distributed throughout the cytoplasm of both the principal and basal cells of the yak epididymis during development (Figure 4A–C). There were no significant differences between the expression levels of SRD5A2 in epithelial cells of the epididymis among the age groups of 1, 2 and 3 years (Figure 5). The negative control did not show any staining (Figure 4A–C insets).

### 3.4. SRD5A2 Expression in Epididymal Epithelial Cells of Mature Yak and Cattle–Yak

**Figure 6:**
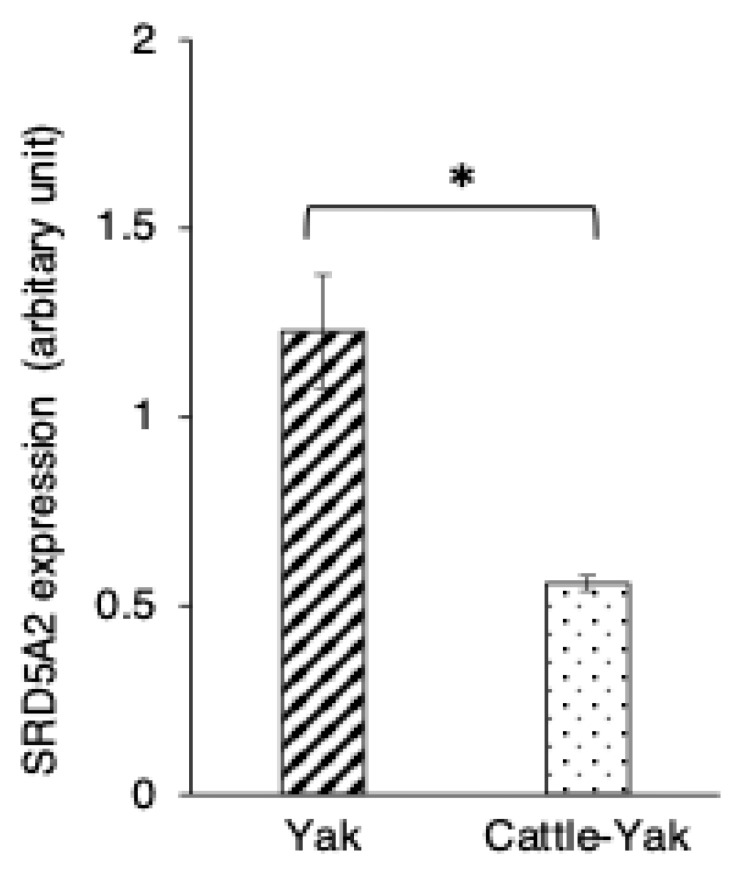
Comparison of 5α-reductase2 expression changes in 2-year-old yaks and 2-year-old crossbred cattle–yaks (F2). 5α-reductase2 expression levels in immunopositive epididymal cells. Bars and error bars represent means ± SEs (yak, *n* = 3; F2, *n* = 3). Asterisk represents significant differences (*p* < 0.05).

SRD5A expression was also detected in the cytoplasm of both principal and basal cells of the epididymis in 2-year-old cattle–yaks (Figure 4D). The expression level of SRD5A2 in the epididymal epithelial cells showed a significantly higher SRD5A2 signal intensity in 2-year-old yaks when compared to in 2-year-old cattle–yaks (*p* < 0.05) (Figure 6). The negative control did not show any staining (Figure 4A,D insets).

## 4. Discussion

Even though cattle–yaks have the same chromosome number as their parents (2*n* = 60) [17], their chromosome morphology is different, which could be the reason for the inactivation or abnormality of gene expression related to spermatogenesis and male sterility [2]. The AR is an important gene that is deficient or has a loss of function in animals with infertility [18]. A previous study showed that the inactivation of the AR gene severely disturbs mature sperm production in AR knockout models [19], suggesting that the AR is necessary for meiosis completion and the transition of spermatocytes to haploid round spermatids [20]. Given that male cattle–yaks are infertile, we hypothesised that the AR might be involved in fertility impairment.

It is believed that male yaks generally reach puberty at 2–3 years of age, and the age of 1 year is considered prepubertal [21]. Since there were no significant differences in AR expression in the epididymal epithelial cells between yaks at age 1, 2 and 3 years of age, it appears that the AR starts to be expressed in the prepubertal yak epididymis. Prepubertal androgen signalling is essential for epididymal development from the intermediate mesoderm and is required for AR expression [22,23]. Our results are in agreement with previous studies in rodents, in which epididymal epithelial cells started to express the AR just prior to birth in mice (embryonic day 19) [24], and there were no discernible differences in AR levels in any cell type in three different areas along the epididymis for both prepubertal and adult rats [25].

Interestingly, unexpected results can be seen in the 2-year-old cattle–yaks, as a significantly higher AR signal intensity in principal cells of the epididymis was observed when compared to that of yaks at the same age. Such increases in AR expression may potentially lead to functional improvements, suggesting that male cattle–yak infertility is not likely due to the impairment of the AR in the epididymal epithelium. However, given that elevated AR expression in Sertoli cells has been reported to disrupt male fertility [26], the potential adverse effects of increased AR expression in the epididymis cannot be excluded. Further studies are needed to clarify its role in male cattle–yak reproduction. Moreover, it should be noted that the current results are not consistent with our previous reports of cattle–yak testes collected in the same season, where decreased AR expression in Leydig cells was found. As the testis is a major regulator of sperm production capacity [27], it has been presumed that infertility in this hybrid species results from AR deficiency in the testicular cells. However, it now appears that other factors are also important, such as SRD5A2 expression.

Testosterone is the most abundant androgen in serum, approximately 3% of which is free and biologically active [20]. Testosterone is introduced into the cell through the cell membrane and can be converted to DHT in the cell by the enzyme SRD5A2, which has a 2–5 times greater binding affinity for AR and a 10-fold greater potency for AR signal induction than testosterone [28]. Therefore, the investigation of SRD5A2 together with AR expression in the epididymal epithelium is of great importance. Our results showed that the expression of SRD5A2 in epithelial cells was stable during development in yaks. The luminal side of epithelial cells often showed dense staining of SRD5A2. Because the testosterone of luminal fluid is supplied from the luminal side of cells, SRD5A2 can convert testosterone to DHT. The expression levels of both the AR and SRD5A2 did not show a change during maturation, suggesting that there is an adequate balance between AR expression and SRD5A2 expression in yaks during development.

In the cattle–yak epididymis, SRD5A2 expression in epithelial cells was significantly lower than in yaks of the same age. Lower SRD5A2 levels are believed to cause a reduction in DHT concentration, thus decreasing the binding affinity of androgen for AR and possibly inhibiting subsequent AR signal transduction during male reproduction. Treatment with 5α-reductase inhibitors in healthy men can cause sperm motility disorders, indicating the importance of DHT in spermatozoa physiology [20,29]. Our results show that male cattle–yak infertility is highly associated with the restriction of SRD5A2 production in the epididymis, which can further interfere with AR signal transduction and sperm physiology. In the case of *Srd5a2* KO mice, increased serum testosterone levels are observed instead of DHT deficiency [30]. One study investigated the mean serum testosterone levels in the hybrid silver fox–blue fox, and the results showed lower concentrations than those in silver and blue foxes [31]. However, in the case of the cattle–yak hybrid, there were normal expression levels of testosterone compared to in the yak [5], suggesting that the expression of SRD5A2 in cattle–yaks seems to be uncorrelated with the plasma testosterone concentration. Thus, hybrid sterility may result from low DHT concentrations induced by the lower expression of SRD5A2 but not testosterone concentration, leading to failure in the maturation of sperm. However, to clarify this phenomenon in hybrid cattle–yaks, further studies will be needed on the interaction between the increased expression levels of AR and decreased expression levels of SRD5A2. Hybrid cattle–yaks show the increase spermatogenic potential by repeated backcrossing; however, fertility is still low. Even if the amount of sperm is small, a high-quality maturation process in the epididymis will provide an increase in fertility. The present study may help establish ways to improve sperm quality through the maturation process.

## 5. Conclusions

The present study indicates that hybrid cattle–yak sterility related to the maturation of sperm may result from a decline in epididymal SRD5A2 production with an increase in AR expression. Although testosterone is believed to regulate the expression of SRD5A2, their mechanism of correlation requires further investigation.

## Data Availability

Data supporting the findings of this study are available from the corresponding author upon reasonable request.

## Abbreviations

The following abbreviations are used in this manuscript:

AR	androgen receptor
SRD5A1	5α-reductase isoform 1
SRD5A2	5α-reductase isoform 2
F1	first generation
F2	second generation
miRNAs	microRNAs
DHT	5α-dihydroxytestosterone
H_2_O_2_	hydrogen peroxide
PBS	phosphate-buffered saline
IgG	immunoglobulin G
BSA	bovine serum albumin
AEC	3-amino-9-ethylcarbazol
ANOVA	analysis of variance
